# Dietary Flavonoids and Human Cognition: A Meta‐Analysis

**DOI:** 10.1002/mnfr.202100976

**Published:** 2022-04-07

**Authors:** Nancy Cheng, Lynne Bell, Daniel J. Lamport, Claire M. Williams

**Affiliations:** ^1^ School of Psychology and Clinical Language Sciences University of Reading, Reading UK

**Keywords:** cognition, effect size, flavonoid, meta‐analysis, polyphenol

## Abstract

Improving cognition is important in all age groups, from performance in school examinations to prevention of cognitive decline in later life. Dietary polyphenols, in particular flavonoids, have been examined for their benefits to cognitive outcomes. This meta‐analysis evaluates the effects of dietary flavonoids on cognition across the lifespan. In January 2020 databases were searched for randomized controlled trials investigating flavonoid effects on human cognition. Eighty studies, comprising 5519 participants, were included in the final meta‐analysis. The global analysis indicates dietary flavonoids induced significant benefit to cognitive performance (*g* = 0.148, *p* < 0.001), with subgroup analyses revealing that cocoa (*g* = 0.224, *p* = 0.036), ginkgo (*g* = 0.187, *p* ≤ 0.001), and berries (*g* = 0.149, *p* = 0.009) yielded the most notable improvements. Significant benefits were observed from chronic studies, in middle‐aged and older adults, and with low and medium doses. The domains of long‐term memory, processing speed, and mood showed sensitivity to flavonoid intervention. This meta‐analysis provides evidence for the positive effects of flavonoids on cognition and highlights several moderating factors. Flavonoid‐based dietary interventions therefore potentially offer a highly accessible, safe, and cost‐effective treatment to help tackle the burden of cognitive decline.

## Graphical abstract

A meta‐analysis reveals positive effects of flavonoids on cognition across the human lifespan. These effects are influenced by flavonoid source, dose, supplementation duration, cognitive health, and age. Benefits are observed following berries, cocoa, and ginkgo supplementation and with low and medium doses. While positive effects are shown in middle‐ aged and older adults, these are more pronounced in cognitively unhealthy groups.

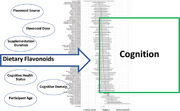

## 1 Introduction

There is increasing consensus that mental health and physical health are interlinked and given that cognitive function is a key contributor to the development and maintenance of good mental health, it will play an important role in overall well‐being. Particular interest has been paid to the impact of diets on cognitive health. Age‐related neurodegenerative disorders such as Alzheimer's disease (AD) and vascular dementia place a significant burden on healthcare provision in an aging population, however epidemiological studies show that diets rich in fruit and vegetables reduce the risk of such disorders and help to improve or maintain cognitive function as we age.^[^ ^1^, ^2^, ^3^, ^4^, ^5^ ^]^ Polyphenols are a group of phytonutrients found in fruits and vegetables that have shown promise as facilitators of cognitive enhancement or neuroprotection. In particular, the polyphenolic subclass of the flavonoids have been extensively studied for their ability to influence cognition and to delay cognitive aging due to their known bioactivity and their high concentrations found in certain food‐types. The biochemistry of flavonoids and proposed mechanisms through which they exhibit their effects on cognition, including activation of neuronal signaling pathways, reduction of neuroinflammation, improvements to vascular function, and interactions with the gut microbiome and neurotransmitter systems, have already been described in detail elsewhere.^[^ ^6^, ^7^ ^]^ Differences in the molecular structure between flavonoid subclasses means that evidence of mechanistic variations are emerging. For example, isoflavones are thought to exert their cognitive effects through their particular affinity for estrogen receptors,^[^ ^8^ ^]^ the degree of hydroxylation around the aromatic rings impacts on the antibacterial properties of flavonoids,^[^ ^9^ ^]^ and there are differential effects on glucose metabolism.^[^ ^10^ ^]^

A number of published studies have investigated the cognitive benefits of the flavonoid family of polyphenols, following supplementation with flavonoid‐rich whole foods or supplements, including berries, cocoa, citrus fruits, tea, soya products, and ginkgo biloba. While the cognitive effects observed following ingestion of any given food cannot necessarily be attributed to a specific polyphenol subclass or subclasses in isolation, flavonoids are the most abundant polyphenol subclass found in foods and beverages and are likely to be present across the interventions investigated in cognitive studies. In contrast, the presence of nonflavonoid polyphenols varies substantially between foods and when characterized, are generally found at lower concentrations than flavonoids.^[^ ^11^ ^]^ The flavonoid content of investigated polyphenol‐rich foods is therefore likely to be responsible for observed neurocognitive responses. Reported effects of flavonoids on cognition have been mixed and suggest that cognitive outcomes may be dependent on several experimental factors which warrant examination. The flavonoid source may be a particularly influential factor, for example Whyte et al.^[^ ^12^ ^]^ found a mixed berries intervention improved measures of executive function whereas Decroix et al.^[^ ^13^ ^]^ found acute cocoa supplementation failed to elicit an effect when also testing executive function in young adults. Duration of flavonoid supplementation is also likely to impact on the detection of cognitive effects, as evidenced by cocoa interventions, whereby chronic treatment in healthy adults typically benefits cognitive performance^[^ ^14^ ^]^ but not following acute treatment in a similar population.^[^ ^15^ ^]^ Characteristics of the population tested, such as age, may modulate the response to flavonoid intervention, for example, Burns et al.^[^ ^16^ ^]^ found that ginkgo was effective at improving long‐term memory in healthy older adults but had no effect on cognitive measures in young adults. Further to this, cognitive health status is also likely to influence cognitive outcomes following flavonoid treatment. This has been indicated by chronic cocoa supplementation, where benefits to cognitive performance were observed in healthy older adults^[^ ^14^ ^]^ but not in older adults with mild cognitive impairment (MCI).^[^ ^17^ ^]^

Several systematic reviews have provided qualitative assessment of the current research showing promise for the positive cognitive effects of flavonoids.^[^ ^18^, ^19^, ^20^, ^21^, ^22^, ^23^, ^24^, ^25^ ^]^ However, the few quantitative meta‐analyses that have been conducted have been limited to specific populations or particular flavonoid‐rich food sources, resulting in conclusions regarding cognitive effects that cannot be generalized more widely to broader populations or applied to other flavonoid‐containing foods.^[^ ^26^, ^27^, ^28^, ^29^, ^30^, ^31^ ^]^ Further, in light of recent reports showing only 33% of the adult population in the UK consumes five or more portions of fruits and vegetables a day,^[^ ^32^ ^]^ such targeted meta‐analyses do not address the wider health benefits of consuming a range of beneficial plant‐based foods in line with government dietary recommendations such as the UK's “5‐ a day” and “eat the rainbow” campaigns.^[^ ^33^ ^]^

Here, we have performed a meta‐analysis of the current literature in order to gain a better understanding of the broader nature and magnitude of flavonoid effects on cognition. Specifically, the primary objective of this meta‐analysis is to investigate whether dietary flavonoids have a positive effect on cognitive outcomes. It is also of interest to understand how flavonoid effects may be modulated. The secondary objectives are therefore to identify the influence of study design (acute or chronic), population characteristics (age and cognitive health status), cognitive domain investigated, and intervention characteristics.

## 2 Experimental Section

The meta‐analysis was preregistered on PROSPERO [CRD42019139022].

### 2.1 Search Strategy

The studies included in the meta‐analysis were identified through a systematic search performed electronically in PubMed, Web of Science, PsycINFO, and Google Scholar databases, from inception to start of January 2020. The keywords: flavonoid, polyphenol, flavanol, flavanone, anthocyanin, isoflavone, berry, blueberry, blackcurrant, acai, goji, cherry, grape, tea, nut, citrus, orange, grapefruit, ginkgo biloba, fruit, cocoa, chocolate, soya in combination with memory, mood, attention, executive function and cognition (including plurals or truncated forms) were used to conduct the search. These search terms were chosen to encompass the subgroups and dietary sources of flavonoids. In addition to the general term of cognition, specific cognitive domains were also used to broaden the search. Filters to exclude nonhuman studies and limit the search to randomized control trials (RCTs) were applied to titles and abstracts. Searches of bibliographies of review articles and searches for additional published articles by major contributors in the field were also performed. Retrieved references were exported from the different databases to EndNote X9 for the removal of duplicate studies. A preliminary pool of relevant RCT trials were identified for consideration against the predetermined inclusion criteria below.

### 2.2 Study Selection

Titles and abstracts of studies retrieved through the search strategy were screened against the following inclusion criteria:

1. randomized trials, parallel or cross‐over design
2. subjected to peer and/or editorial review
3. human subjects – any health state, age, or other demographic characteristics 
4. flavonoids content of intervention specified
5. acute or chronic trials 
6. appropriate placebo‐controlled design (or inclusion of a suitable control condition – see modifications below)
7. studies that had utilized one or more cognitive tasks
8. sufficient data to calculate effect size(s)
9. written in English

If the above details could not be established from the title or abstract, then the full text was used to assess a study against the inclusion criteria. Studies that did not meet these criteria were excluded. Two investigators (N.C. and L.B.) independently completed screening of studies for inclusion. In the event of any discrepancies between study selections, a resolution was achieved through discussion to reach a consensus or referred to a third reviewer (D.L. or C.W.) if a consensus was not initially achieved.

#### 2.2.1 Modifications to Inclusion Criteria

During the study selection process, it became apparent that most studies compared one or more flavonoid supplement(s) with a control, rather than an inert placebo. A consensus was reached between two investigators that the term “control” better reflected the study designs of interest. The minor modification to criterion 6 from “appropriate placebo‐controlled design” to “appropriate control condition employed” was made.

Studies that investigated a combination of flavonoid and nonflavonoid active treatments which did not isolate the cognitive effects of the flavonoid component were highlighted for discussion. Two investigators reached the conclusion that these studies did not specifically answer the research question of whether flavonoids had a positive effect on cognition. “Investigation of flavonoid active treatments only” was therefore added to the inclusion criteria.

### 2.3 Data Extraction

Full‐text copies of the selected studies were obtained, and data extracted by two independent investigators (N.C. and L.B.) using a standardized Excel form. Any discrepancies were resolved by discussion to reach consensus, with a third reviewer (D.L. or C.W.) consulted if a consensus could not be achieved. In cases where there was insufficient data present in the paper for analysis, authors were contacted via email using the contact information supplied on the paper. If no reply was received, then the study was excluded. To inform the primary objective of whether dietary flavonoids had a positive effect on cognitive outcomes the following data were extracted from each study for a global analysis: first author's name, year of publication, number of participants, cognitive scores (means and standard deviations/standard errors), and correlations between repeated data. For the purpose of the secondary objective, to identify the influence of study design, population characteristics, cognitive domain investigated and intervention characteristics, the following data were extracted from each study for moderator analysis: study design—acute/chronic, parallel/crossover; type of nutritional intervention—dietary source; dose of administered intervention (expressed as flavonoid or polyphenol content); treatment duration; age and health status of study participants; and cognitive task type/domain. Meta‐analyses commonly select either parallel or crossover design studies for review both for compatibility purposes and to limit heterogeneity was acknowledged. However, in order to successfully answer the main research question posed by this review, the review has been as inclusive as possible and incorporated both study designs. Inclusion of both types of study designs permitted the comprehensive assessment of the current literature since both designs contributed to the research question posed here. Exclusion of either type would have reduced the number of studies significantly and limited the generalizability of the results. The heterogeneity of the effect sizes between parallel and crossover studies was computed as part of this meta‐analysis to assess their impact on the overall estimated effect size.

### 2.4 Data Coding

Cognitive tasks employed in the selected studies were extracted and then coded according to the Cattell–Horn‐Carroll (CHC) classification^[^ ^34^, ^35^ ^]^ in order to investigate whether any specific cognitive domains were selectively responsive to the effects of flavonoids. Individual studies often reported data for multiple cognitive outcomes, across multiple cognitive domains. In such cases, all data were included in the meta‐analysis. Where multiple timepoints were reported in a single study, all timepoints were also included in the data extraction. Coding of study design, flavonoid source, flavonoid dose, duration of supplementation, age of participant, participant cognitive health status, were described under Section 2.7. All data were collated in a standardized form in Excel.

### 2.5 Risk of Bias (Quality) Assessment

Risk of bias/quality of the included studies was assessed by two investigators (L.B. and N.C.) using the Cochrane “risk of bias tool” for randomized trials^[^ ^36^ ^]^ as part of the data extraction procedure recorded using Excel. Any discrepancies were resolved through discussion. If agreement could not be reached, a third investigator was consulted (see Results section for outcomes).

### 2.6 Data Synthesis

The software package ``Comprehensive Meta‐analysis’’ (CMA for Windows, version 3, Biostat, Englewood, NJ 2013, USA) was used to conduct a quantitative meta‐analysis of the aggregate data. Due to the varied methodology employed by the included studies, a random‐effects model was used in the global analysis. Hedge's *g*, which corrects for bias in small sample sizes,^[^ ^37^ ^]^ and 95% confidence intervals were calculated as an estimate of the summary effect size for each study, using preintervention and postintervention means and standard deviations (SD) and sample sizes for treatment and placebo groups. Directions of effects were coded, whereby a positive value represented a positive effect of flavonoid, and a negative value represented a negative effect on an outcome measure. This involved reverse coding for some measures, such as reaction times, where a positive value represented a negative effect on cognition. For each study, correlations between pre‐ and postintervention scores were estimated by taking a mean average of the within‐control and within‐treatment correlation coefficients (Pearson *r*). As this information was generally not available in the published articles, these coefficients were often determined by calculation using the formula below (where pre = preintervention, post = postintervention, and diff = change from baseline difference):

(1) $$r=\left(S{D^{2}}_{\mathrm{pre}}+S{D^{2}}_{\mathrm{post}}-S{D^{2}}_{\mathrm{diff}}\right)/\left(2\times SD_{\mathrm{pre}}\times SD_{\mathrm{post}}\right)$$

An average of these prepost intervention correlations was used as an estimate for studies with insufficient data to complete this calculation, in accordance with meta‐analysis guidelines described in the Cochrane Handbook.^[^ ^38^ ^]^ In studies where only postintervention data was reported, means, SDs, and sample sizes were collated for treatment and control groups. Additionally, for studies where only change from baseline (CFB) data was available, postintervention SDs were estimated by calculation using the formula below (where post = postintervention, diff = change from baseline, and *r* = average prepost correlation):

(2) $${\mathrm{SD}}_{\mathrm{post}}={\mathrm{SD}}_{\mathrm{diff}}/\sqrt{\left(2\left(1-r\right)\right)}$$

The above estimates allowed conversion of multiple types of data for standardization by postintervention SDs, which enabled direct comparisons of effects in the meta‐analysis. Computation of Hedge's *g* was performed using CMA software. For most studies, data were entered in mean and SD format. For five studies, data were entered directly as effect size values (Cohen's *d*) where this was the only data available in the paper^[^ ^39^ ^]^ or where data in a mixture of formats were obtained and required conversion to a single format for input into the CMA software.^[^ ^40^, ^41^, ^42^, ^43^ ^]^

Where studies reported multiple experiments with different participant groups, these experiments were treated as separate studies for entry into the meta‐analysis.^[^ ^16^, ^44^, ^45^, ^46^ ^]^ Where studies reported outcomes for multiple doses or treatment interventions but used the same control group for each comparison, all intervention data were averaged to provide a single entry. In these circumstances, it was considered best practice to combine all relevant experimental intervention groups of the study into a single group, and to combine all relevant control intervention groups into a single control group.^[^ ^38^ ^]^

Heterogeneity between studies was investigated using a combination of the *Q* statistics and *I* ^2^ statistic according to Borenstein et al.^[^ ^37^ ^]^ For clarity, the *Q* statistic with its associated *p*‐value indicates if existing heterogeneity is statistically significant, while the *I* ^2^ statistic further indicates the extent of heterogeneity present whereby an *I* ^2^ value of greater than 50% indicates substantial heterogeneity. Visual inspection of the symmetry of the funnel plot and Fail‐safe N were used to assess publication bias.

### 2.7 Moderator Analysis

Further analyses of the included studies were conducted according to the following moderators: flavonoid source, duration of supplementation, age of participant, and cognitive health status of participant. Mixed effects models were used to compare effect sizes of moderator subgroups.

The studies were divided into the following flavonoid source subgroups; berry, citrus, cocoa, ginkgo, pine bark, soya, tea, or other; and the extent to which these subgroups explained any heterogeneity in the global analysis was examined. Duration of supplementation was categorized to correspond with the timeframe of acute studies and significant increases in participant burden into the following categories: up to and including 24 h; greater than 24 h, up to and including 6 weeks; greater than 6 weeks, up to and including 3 months; and greater duration than 3 months.

Age was categorized according to key physical, social, and cognitive developmental changes across the lifespan^[^ ^47^, ^48^ ^]^ into child (0–12 y), adolescents (13–18 y), young adult (18–39 y), middle aged adult (40–59), and older adult (60y+). Where the age ranges of study subjects straddled two or more subgroups, the mean age reported in the paper was used to determine the subgroup classification. For the cognitive health factor analysis, studies were divided into healthy subjects and cognitively unhealthy. Participants with a diagnosis of a condition associated with cognitive deficit which was likely to negatively impact on their performance in cognitive tasks, were classified as cognitively unhealthy. This included participants with mild cognitive impairment, dementia, and Down's Syndrome.

For some moderators, including flavonoid dose, design (acute or chronic), and cognitive domain, individual studies often reported results for multiple subgroups within a moderator category. Separate meta‐analyses were therefore performed with data for each subgroup entered individually, rather than a comparative analysis which would lead to the exclusion of these studies.

For the “Dose meta‐analysis,” reported flavonoid doses were categorized as Low, Medium, or High based on habitual dietary averages. Global patterns of flavonoid consumption^[^ ^49^, ^50^, ^51^, ^52^ ^]^ were used to create a scale of flavonoid intake according to dietary source against which research doses were mapped. The categories of flavonoid doses according to source are outlined in **Table** **1**.

**Table 1:** Categorization of dose ranges for each flavonoid source based on typical habitual intake

	Dose range [mg]		
Flavonoid source	Low	Medium	High
Cocoa	0–349	350–699	700+
Citrus	0–74	75–149	150+
Tea	0–424	425–849	850+
Ginkgo	0–39	40–79	80+
Berry	0–349	350–699	700+
Soya	0–69	70–139	140+
Pine bark	0–249	250–499	500+
Other	N/A	N/A	N/A

Acute and chronic studies were individually assessed. Acute studies were classed as those with a single dose and a testing duration of 24 h or less. Studies testing at timepoints beyond 24 h, with repeated daily supplementation, were classed as chronic studies.

Cognitive domains were divided into subgroups according to CHC classifications: word fluency, acquired knowledge, fluid reasoning, long‐term memory encoding and retrieval, processing speed, working memory/short‐term memory, visuospatial ability, reaction/decision speed, subjective mood (where subjects self‐rated how they were feeling), and unclassified tasks with multiple parts that straddled many domains (e.g., Mini mental state examination MMSE).^[^ ^35^ ^]^

## 3 Results

**Figure 1:**
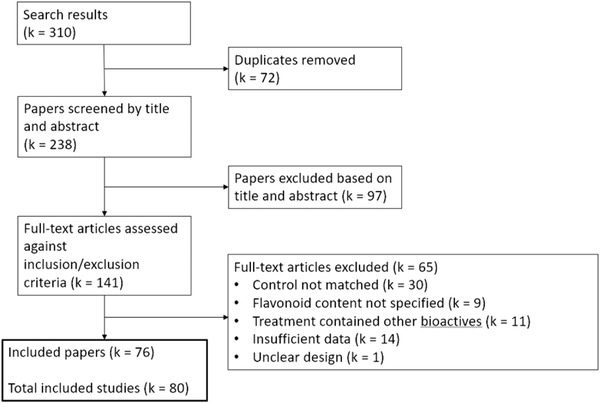
Flow diagram of the literature selection process outlining identification of included studies for meta‐analysis of the effect of flavonoids on cognition.

Our preliminary literature search yielded 310 studies. Screening against the predetermined eligibility criteria identified 76 papers for further review,^[^ ^12^, ^13^, ^14^, ^15^, ^16^, ^17^, ^24^, ^39^, ^40^, ^41^, ^42^, ^43^, ^44^, ^45^, ^46^, ^53^, ^54^, ^55^, ^56^, ^57^, ^58^, ^59^, ^60^, ^61^, ^62^, ^63^, ^64^, ^65^, ^66^, ^67^, ^68^, ^69^, ^70^, ^71^, ^72^, ^73^, ^74^, ^75^, ^76^, ^77^, ^78^, ^79^, ^80^, ^81^, ^82^, ^83^, ^84^, ^85^, ^86^, ^87^, ^88^, ^89^, ^90^, ^91^, ^92^, ^93^, ^94^, ^95^, ^96^, ^97^, ^98^, ^99^, ^100^, ^101^, ^102^, ^103^, ^104^, ^105^, ^106^, ^107^, ^108^, ^109^, ^110^, ^111^, ^112^, ^113^ ^]^ comprising 80 experimental studies. A summary of the study selection process is outlined in **Figure** **1**.

**Figure 2:**
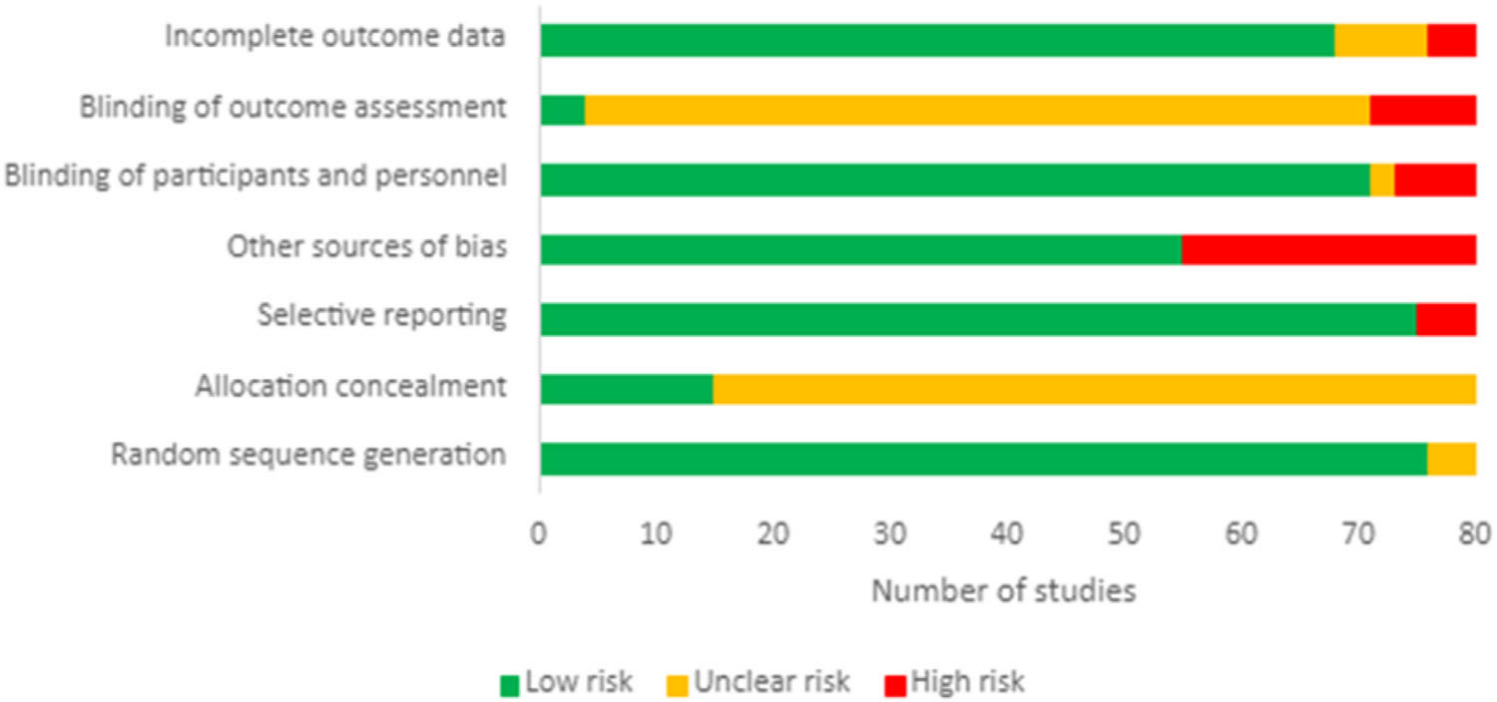
Methodological quality summary: review authors’ judgments about each methodological quality item from the Cochrane Risk of Bias tool for each study included in the meta‐analysis.

The overall quality of the studies was high when assessed against the Cochrane Risk of Bias Tool (**Figure** **2**). While sources of bias were identified in the methods and reporting of results, there was an overall low risk that the true effects of flavonoids on cognitive performance had been affected by bias from the included studies. For instance, minor sources of bias were identified in relation to blinding of participants and personnel where seven studies were single‐blinded,^[^ ^12^, ^46^, ^54^, ^66^, ^90^, ^109^, ^113^ ^]^ incomplete outcome data was reported in four studies,^[^ ^16^, ^40^, ^89^, ^106^ ^]^ and nonblinding of outcome assessments was apparent in nine studies.^[^ ^12^, ^46^, ^54^, ^65^, ^66^, ^90^, ^105^, ^109^, ^113^ ^]^ Selective reporting was detected in five studies, including omission of nonsignificant results,^[^ ^79^, ^84^, ^85^ ^]^ reporting of phase 1 only in a crossover study^[^ ^77^ ^]^ and reporting of mood data excluding cognitive results.^[^ ^46^ ^]^

**Figure 3:**
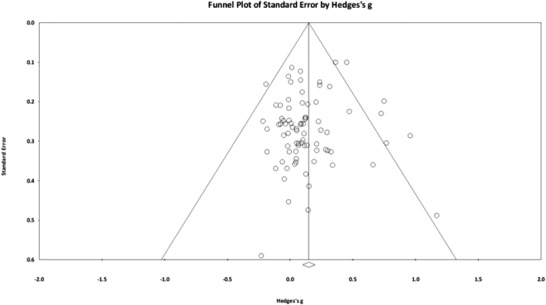
Funnel plot for the cognitive effects of flavonoids showing no publication bias.

Twenty‐four studies included “other” sources of bias mainly in relation to the appropriateness of the placebos administered, typically these included ingredients which were not matched to the treatment with unclear effects on measured outcomes. Eleven studies reported the addition of either natural or artificial coloring,^[^ ^24^, ^57^, ^59^, ^63^, ^70^, ^71^, ^73^, ^79^, ^83^, ^89^, ^91^ ^]^ while one study used coloring in both the treatment as well as the placebo.^[^ ^109^ ^]^ One study used a placebo containing fructo‐oligosaccharides,^[^ ^108^ ^]^ six studies used placebos which themselves contained flavonoids.^[^ ^15^, ^17^, ^68^, ^82^, ^93^, ^105^ ^]^ One study did not report final *n* values for results of dependent variables where subjects had difficulties with the cognitive tasks involved.^[^ ^41^ ^]^ Finally, four studies reported chronic data where measurements were in fact recorded in the immediate postprandial period following the final chronic dose.^[^ ^44^, ^45^, ^72^, ^97^ ^]^ Visual inspection of the funnel plot showed no evidence of publication bias (**Figure** **3**). This was confirmed by a Fail‐safe *N* value of 448.

### 3.1 Global Effect of Flavonoids on Cognition

**Figure 4:**
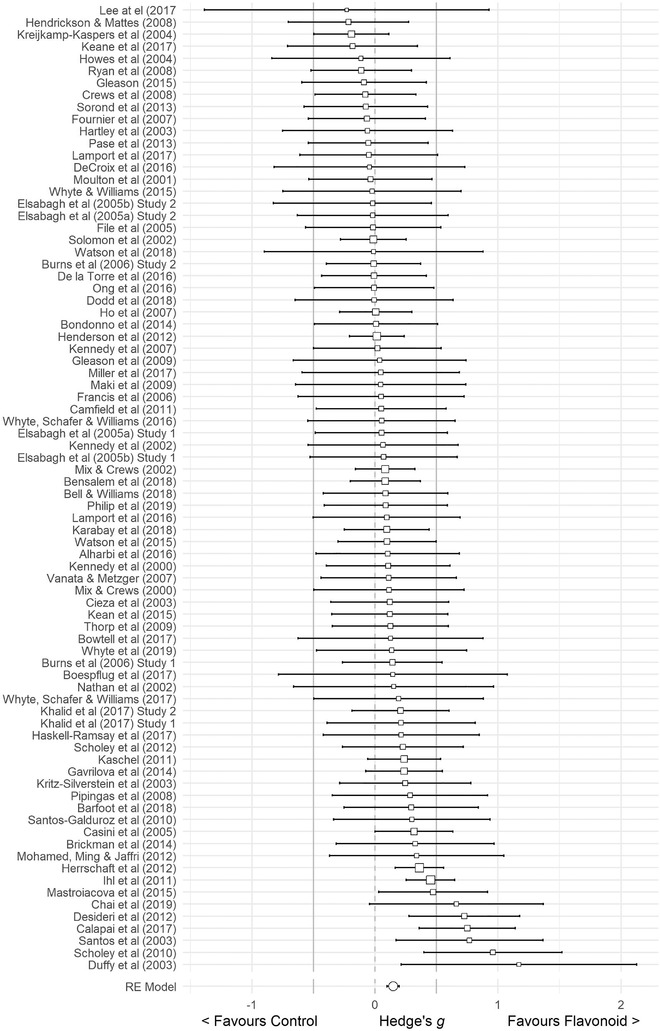
Forest Plot of studies investigating the effects of flavonoid intervention on cognition, depicting Hedge's *g* with associated 95% confidence intervals.

Eighty studies were included in this meta‐analysis, with sample sizes ranging from *n* = 10^[^ ^110^ ^]^ to *n* = 410.^[^ ^75^ ^]^ Data from 5519 participants were pooled in our global meta‐analysis. Using a random effects model, the computed effect size, Hedge's *g*, was 0.148 (SE = 0.025, 95% CI 0.098–0.198, *Z*‐value = 5.825, *p* < 0.001: **Figure** **4**) in favor of flavonoids, without significant heterogeneity (*Q* = 79.451, *df* = 79, *p* = 0.465, *I* ^2^ = 0.567%). This indicates that flavonoid supplementation has a significant positive effect on measures of cognitive performance. Despite mixed findings from individual studies, the reported effect sizes across the literature are not significantly different from each other.

### 3.2 Moderator Analyses

Full results for all moderator analyses can be found in tabulated form in the accompanying Supplementary Information, but the main findings are summarized below.

#### 3.2.1 Comparative Analyses

**Figure 5:**
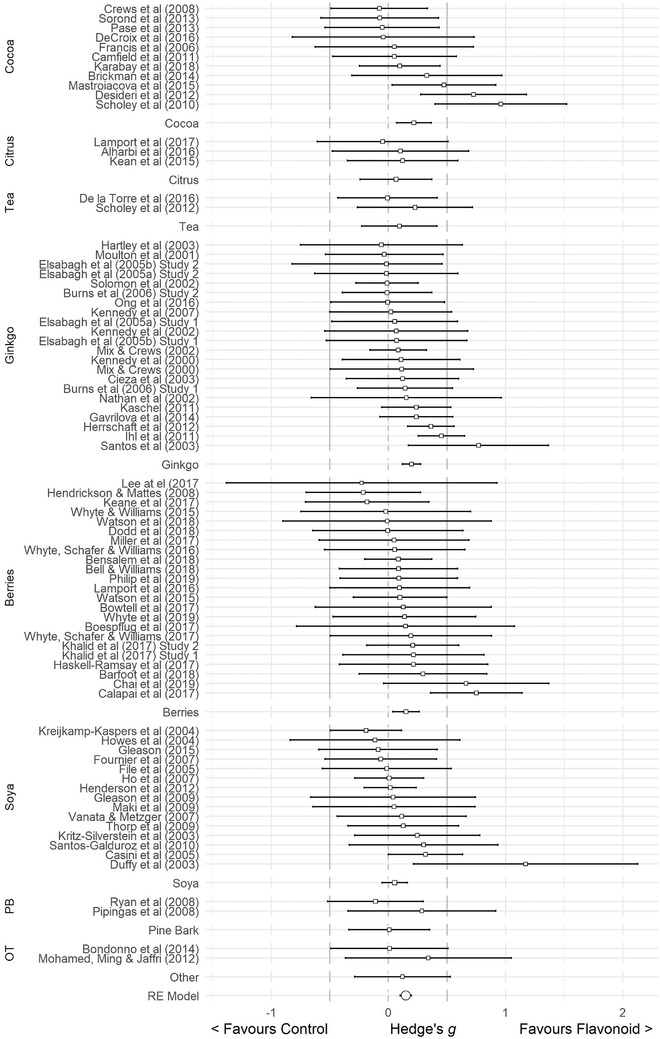
Forest Plot of the effects of flavonoid intervention on cognition according to flavonoid source, depicting Hedge's *g* with associated 95% confidence intervals.

*Flavonoid source*: The results of the mixed‐effects model indicated significant benefits to cognition in favor of berries, ginkgo, and cocoa, with the largest effects seen following cocoa interventions (*k* = 11, *g* = 0.224, 95% CI = 0.014–0.434, *p* = 0.036), followed by ginkgo (*k* = 22, *g* = 0.187, 95% CI = 0.103–0.271, *p* ≤ 0.001) and then berries (*k* = 23, *g* = 0.149, 95% CI = 0.038–0.261, *p* = 0.009). Berries, ginkgo, and cocoa studies represent most of the included studies. Collectively these three subgroups comprise 56 of the 80 studies. In general, the smaller subgroups, citrus (*k* = 3), pine bark (*k* = 2), tea (*k* = 2), and other (k = 2) subgroups did not yield significant benefits to cognition although Hedge's *g* was positive in all cases (favoring flavonoid intervention). Soya was one of the larger subgroups (*k* = 15), but studies were notably focussed on postmenopausal women. The overall difference between subgroups of flavonoid sources was not significant, *Q*(7) = 5.109, *p* = 0.647. **Figure** **5** depicts the results of the flavonoid source analysis in a forest plot. The largest effect size was observed for the cocoa subgroup, but this was still small in overall magnitude.

*Duration of Supplementation*: Acute studies were categorized in the duration of supplementation analysis as “up to and including 24 hours.” As per the acute/chronic analysis, positive effects on cognition that were near to significance were demonstrated here (*k* = 31, *g* = 0.094, 95% CI = −0.002–0.190, *p* = 0.055). The analysis of supplementation duration further revealed that chronic treatment with flavonoids for less than 6 weeks did not produce a significant effect on cognition (*p* = 0.912). However, only three studies fell into this category and so this analysis may lack power. Treatment for greater than 6 weeks up to and including 3 months (*k* = 31) resulted in significant effects on cognition (*p* = 0.001). Increasing the duration of the intervention to greater than 3 months (*k* = 19) yielded a similar significant effect (*p* = 0.008). Heterogeneity between the supplementation duration subgroups was not significant *Q*(3) = 1.604, *p* = 0.659.

*Age*: The Age subgroup analysis showed a small, yet significant benefit to cognition, in middle‐aged (*k* = 22, *p* = 0.037) and older adults (*k* = 29, *p* = 0.001). Treatment with flavonoids did not show significant benefits in children (*k* = 5, *p* = 0.172) and young adults (*k* = 24, *p* = 0.105). The number of studies in children is notably sparse and it is noteworthy that, of the five studies involving children as participants that were analyzed here, all were acute design and investigated blueberry supplementation. Further to this, there is an absence of studies conducted in the adolescent age group. However, young adults are well‐represented by 24 studies in this meta‐analysis. Heterogeneity of the effects between age subgroups was not statistically significant *Q*(3) = 1.605, *p* = 0.658.

*Cognitive Health Factor*: Subgroup analysis revealed significant benefits of flavonoids on cognition in both healthy (*p* ≤ 0.001) and cognitively unhealthy subjects (*p* ≤ 0.001). These effects were found to be significantly heterogenous *Q*(1) = 5.319, *p* = 0.021, with larger effect sizes observed in cognitively unhealthy participants versus healthy participants (*g* = 0.306 and 0.103, respectively). The positive effects in the cognitively unhealthy subgroup were observed despite the small number of studies analyzed (*k* = 8). Interestingly, of the eight studies in the cognitively unhealthy subgroup, seven of these involved older adult participants.^[^ ^11^, ^48^, ^60^, ^62^, ^66^, ^69^, ^82^ ^]^ The one exception was a study in young adults with Down's Syndrome.^[^ ^56^ ^]^ Four of the older adult studies were conducted in MCI patients,^[^ ^11^, ^48^, ^60^, ^82^ ^]^ the remaining three studies investigated flavonoid effects in patients with Alzheimer's disease or vascular dementia.^[^ ^62^, ^66^, ^69^ ^]^

*Parallel or Crossover Group Design*: We analyzed the effect sizes as a function of the study design (parallel or crossover). Both designs showed significant positive effects of flavonoids on cognition. The difference in effect sizes between the two study design types was nonsignificant, *Q*(1) = 0.075, *p* = 0.785. This clearly shows that the observed benefits to cognitive performance following flavonoid intervention occur irrespective of the study design employed.

#### 3.2.2 Individual Analyses

For moderators where some or all studies reported results for multiple subgroups, comparator analyses were not possible. Results of the analyses of individual subgroups are presented here.

*Acute or Chronic Design*: The acute/chronic design meta‐analyses revealed studies employing chronic flavonoid interventions showed a small but significant benefit to cognition (*p* ≤ 0.001) and studies which involved acute flavonoid supplementation showed an effect approaching significance (*p* = 0.055). Despite reasons of financial burden and patient commitment given as constraints to the running of longer‐term trials, a larger proportion of the analyzed studies were chronic in design. Thirty‐one acute studies and 51 chronic studies were analyzed here.

*Dose of flavonoid*: Many of the reviewed studies investigated the effects of a low (46 studies) or medium flavonoid dose (31 studies). Only 13 studies used a high‐dose flavonoid intervention, representing 561 subjects. The Dose meta‐analyses revealed studies employing low and medium doses of flavonoids showed significant benefits on cognition, (*p* = 0.013 and *p* ≤ 0.001 respectively). Studies which tested high flavonoid doses showed no significant effects overall (*p* = 0.181).

*Cognitive Domain*: The Cognitive Domain analysis showed significant effects of flavonoid intervention in three cognitive domains. These were long‐term memory (*p* = 0.014), processing speed (*p* = 0.008), and subjective mood (*p* = 0.006) which are among the largest subgroups that were analyzed (*k* = 55, 52, and 34 respectively). All other cognitive domains; word fluency (*k* = 24), acquired knowledge (*k* = 14), fluid reasoning (*k* = 19), working memory/short‐term memory (*k* = 46), visuospatial ability (*k* = 16), and reaction/decision speed (*k* = 24), failed to yield significant effects. The potential impact of the number of studies (*k*) included in the subgroup analysis for each cognitive domain is explored in the discussion.

## 4 Discussion

To our knowledge this is the first meta‐analysis to assess data obtained from randomized control trials covering a range of dietary flavonoids across multiple populations, and their effects on cognitive function, encompassing a variety of cognitive domains across the human lifespan. The results of our global analysis showed that flavonoid supplementation has a significant (though small) positive effect on cognition. Funder and Ozer^[^ ^114^ ^]^ suggest that small effect sizes in psychological measures may have important impact in the longer term, particularly when considering health outcomes where effects are likely to be cumulative, such as cognitive health^[^ ^115^ ^]^ as observed here. Thus, the small global effect of flavonoids on cognition (*g* = 0.148) has potential significance. Importantly, the overall assessment of a low risk of bias in the included studies and minimal publication bias gives confidence in our results.

The analysis of moderators also revealed several interesting findings. The effect of flavonoids on cognitive measures was shown to be influenced by flavonoid source, where significant positive effects were observed with berries, ginkgo, and cocoa. Other intervention characteristics which demonstrated moderating effects were dose and duration of supplementation. Here, low and medium flavonoid doses showed significant benefits to cognition with chronic treatments of 6 weeks and longer showing significant effects on cognitive performance and acute interventions yielding near significant effects. Additionally, with respect to participant factors, both age and cognitive health status were found to moderate the cognitive response to flavonoid supplementation. Significant benefits were measured in middle‐aged and older adults, which is likely to be driven by the influence of cognitive health status, whereby more pronounced effects were seen in cognitively unhealthy compared with healthy participants. Our results also revealed the cognitive domains of long‐term memory, mood and processing speed to be sensitive to flavonoid treatment.

### 4.1 Flavonoids and Cognition

The positive result of our global analysis is consistent with meta‐analyses by Poti et al.^[^ ^31^ ^]^ and Ammar et al.^[^ ^26^ ^]^ Poti et al. showed significant effects of polyphenol supplementation on the Wechsler Adult Intelligence Scale (WAIS) – Block design, Rey's auditory verbal learning task (RAVLT) immediate recall and Trail‐Making Task B (TMT‐B), reported in test score metrics, in older adults.^[^ ^31^ ^]^ Ammar et al. observed improvements in Simple Reaction Time and Serial 7s with moderate effect sizes (SMD = ‐0.926 and 1.467 respectively) and Mental Fatigue (very large effect size of −3.521) in young and middle‐aged adults.^[^ ^26^ ^]^ The specificity of the outcome measures included in these meta‐analyses could explain the larger effect sizes seen in comparison to our global analysis. Other differences in methodology, namely inclusion of nonflavonoid polyphenols, such as resveratrol, and different populations of interest, may also have contributed to the larger effect size estimates seen by Ammar et al.^[^ ^26^ ^]^ In contrast, another recent meta‐analysis by Ammar et al.^[^ ^27^ ^]^ did not find any significant effects of polyphenols on cognition in older adults, which reviewed performance in TMT‐A and TMT‐B only. Both Poti et al.^[^ ^31^ ^]^ and Ammar et al.^[^ ^27^ ^]^ assessed performance in TMT‐B in older adults. However, Poti et al. investigated the effects of polyphenols in healthy individuals as well as MCI patients,^[^ ^31^ ^]^ whereas Ammar et al. limited their meta‐analysis to healthy subjects.^[^ ^27^ ^]^ In general, faster TMT‐B completion times were reported for healthy older adults^[^ ^26^ ^]^ compared with older adults including those with MCI.^[^ ^31^ ^]^ Although clear ceiling effects were not observed, TMT may place insufficient cognitive demand on healthy subjects to detect cognitive changes following polyphenol supplementation. This is in keeping with evidence indicating flavonoids are most effective at improving cognition in more cognitively challenging situations.^[^ ^24^, ^112^ ^]^ This difference in population characteristic is likely to explain the discrepancy in review outcomes. Indeed, cognitive health status was examined in our cognitive health subgroup analysis and suggests that the presence of cognitive deficits provide greater potential for measurable improvements in cognitive performance.

The small effect size observed in our global meta‐analysis is in keeping with the magnitude of effect sizes demonstrated by other nutrients and food components. For example, in a meta‐analysis by Suh et al.,^[^ ^116^ ^]^ over 3 months of vitamin B (folate, vitamin B6, and vitamin B12) was associated with improved global cognition (SMD = 0.18) and episodic memory (SMD = 0.09) in middle‐aged and older adults. Vitamin B is thought to reduce homocysteine levels, beyond which multiple biological mechanisms of action could explain the connection it has with improved cognitive performance, including vascular mechanisms, prevention of neuronal apoptosis, and epi‐genetic modifications.^[^ ^117^ ^]^ Similarly, a range of vascular, metabolic, and neurochemical mechanisms of action have been proposed for flavonoids and their benefits to cognition.

Our findings have important implications for the interpretation of existing flavonoid research and for the design of future clinical trials investigating the impact of flavonoids on cognition. The common practice of using medium to large effect size estimates to establish subject sample size is likely to lead to underpowering of trials. Appropriate sampling should in fact consider a small expected effect size. Indeed, study sample size and other research best practices in the wider field of nutrition and cognition have received commentary, including by Brydges and Gaeta^[^ ^118^ ^]^ who found publication bias in the blueberry and cognition studies reviewed by Hein et al.^[^ ^119^ ^]^ However, it should be noted that the analysis by Brydges and Gaeta used *p*‐values drawn from only the key findings from the individual studies and were not representative of all reported results in the original papers. A subsequent reanalysis by Whyte et al. accounting for all the dependent variables reported in the original source papers found no evidence of publication bias.^[^ ^120^ ^]^ Importantly, in this current meta‐analysis the computed Fail safe *N* value was 448, meaning a further 448 nonsignificant studies would be required for alternative conclusions in relation to our primary research objective to be drawn. Reassuringly, the risk of publication bias affecting the results of our meta‐analysis is very low.

In addition to the global analysis, our subgroup analyses also revealed some interesting findings with regard to moderators of flavonoid induced benefits to cognition that warrant further discussion.

#### 4.1.1 Moderators of flavonoid effects

The subgroup analysis of flavonoid sources showed significant effects for berries, ginkgo, and cocoa, whereas citrus, soya, pine bark, and tea revealed no benefit to cognition. It is widely accepted that the bioavailability of flavonoids and their metabolites is a key determinant of their efficacy in relation to pharmacological effects. It is therefore reasonable to expect the bioavailability of the flavonoid subclasses (reviewed by Di Lorenzo et al.^[^ ^121^ ^]^), to contribute to their effectiveness in relation to cognitive measures. In light of this, variations in the cognitive effects according to dietary flavonoid source could be attributed to their flavonoid profile and predominant flavonoid subclass(es); such as flavan‐3‐ols in cocoa, anthocyanins in berries and flavanones in citrus fruit.^[^ ^122^ ^]^ Indeed, Ammar et al.^[^ ^27^ ^]^ suggested differences in individual study results observed with soya and blueberry were linked to the bioavailability of isoflavones and anthocyanins respectively. The nonsignificant effect of tea demonstrated in the current meta‐analysis is in‐keeping with the low bioavailability of galloylated tea catechins. However, our subgroup analysis findings cannot solely be explained by flavonoid bioavailability, especially as absorption rates for anthocyanins are reported to be low, but isoflavone absorption rates are high.^[^ ^123^, ^124^ ^]^ Here, we observed significant cognitive effects for anthocyanin‐rich berries, whereas isoflavone‐rich soya interventions were not found to be effective. The number of included studies per subgroup is likely to be a relevant factor. Significant effects were seen with the largest subgroups; ginkgo (*k* = 22), berries (*k* = 23), and cocoa (*k* = 11). Apart from soya (*k* = 15), benefits did not achieve statistical significance for the smallest subgroups; citrus (*k* = 3), pine bark (*k* = 2), tea (*k* = 2), and others (*k* = 2), possibly suggesting a lack of statistical power. However, in addition to the number of studies, it is also important to consider the mechanisms of action of these flavonoid compounds within the central and peripheral nervous system. As with pharmacological effects in general, some flavonoid types may impact cognitive function more effectively than others through mechanistic differences (as described in the Introduction), or only be effective in specific populations. However, it was beyond the scope of this current meta‐analysis to investigate this.

For ginkgo biloba, our meta‐analysis revealed a significant benefit to cognition. This may reflect the fact that many of these studies focussed on older or cognitively impaired populations. Indeed, previous meta‐analyses which have focussed on dementia and Alzheimer's disease patients have similarly reported benefits to cognition,^[^ ^125^, ^126^ ^]^ particularly in response to supplementation with 240 mg of the standardized extract EGb 761.^[^ ^127^, ^128^ ^]^ In healthy subjects, however, Laws et al.^[^ ^129^ ^]^ observed no improvements to memory, executive function, nor attention. These inconsistencies are likely due to health status differences in the populations investigated, where cognitively impaired subjects have greater scope for improvement and response to flavonoid treatment. Indeed, this is an outcome we have demonstrated in the current health factor analysis that revealed much greater effect sizes in cognitively unhealthy subjects.

The lack of a significant effect with soya supplementation observed here is contrary to the findings of two previous meta‐analyses which assessed the effects of soya and soy isoflavones on cognition. These found positive effects in postmenopausal women (SMD = 0.08)^[^ ^29^ ^]^ as well as younger men and women (SMD = 0.19),^[^ ^30^ ^]^ which demonstrated similarly small effect sizes. Discrepancies in our results may be due to differences in study selection criteria. Our strict inclusion criteria meant that some studies included in these two previous meta‐analyses, were excluded from our meta‐analysis based on the use of controls containing nonflavonoid active ingredients. Cheng et al.^[^ ^29^ ^]^ limited their meta‐analysis to postmenopausal women, whereas our review included all populations. Cui et al.^[^ ^30^ ^]^ excluded red clover as a source of soya isoflavones, whereas our inclusion criteria permitted its inclusion. These methodological differences may therefore account for some of the differences in findings.

The analysis of acute and chronic designs showed significant effects of chronic flavonoid intervention on cognitive performance, while the acute effect only approached significance. Further to this, our Duration of Supplementation subgroup analysis showed significant effects of flavonoid supplementation for periods greater than 6 weeks up to 3 months, and for periods longer than 3 months. This result suggests that for chronic supplementation, at least 6 weeks of flavonoid treatment is required for cognitive benefits to manifest and further corroborates the cumulative nature of flavonoid effects. Indeed, while much focus has been placed on the metabolism of flavonoids in the 24 h immediately after consumption, with identification of associated peak plasma levels (see review by Di Lorenzo et al.^[^ ^121^ ^]^), our findings point to the importance of assessing flavonoid metabolism over an extended period. Although longer trial durations have financial and recruitment implications, when planning trials researchers should consider a minimum of 6 weeks of supplementation, to identify extended time‐course effects and to allow sufficient time for detectable cognitive changes to occur.

Our observation of stronger improvements to cognition following chronic relative to acute supplementation may be partly explained by the rate at which bidirectional effects between flavonoids and the gut microbiota occur. Flavonoids can act as prebiotics to enhance the growth and establishment of beneficial strains of bacteria present in the gut including bacteria from the Bifidobacteriaceae and Lactobacillaceae families.^[^ ^130^, ^131^ ^]^ In turn, biotransformation by metabolism, depolymerization and deconjugation of unabsorbed flavonoid molecules by the gut microbiota occurs in the colon to promote bioavailability of active metabolites known to elicit both general and cognitive health effects (see reviews for proposed flavonoid mechanisms of action^[^ ^6^, ^7^, ^134^ ^]^). The gut microbiota further interacts with the central nervous system through the production of neurotransmitters, for example γ‐amino butyric acid (GABA) by species of Lactobacillus and Bifidobacterium^[^ ^132^ ^]^ (see detailed review of flavonoid and gut microbiota relationship).^[^ ^133^ ^]^ Improved cognitive performance shown for flavonoid treatments of 6 weeks and longer likely reflect the time required for changes to the gut microbiota to take place and for associated increases in flavonoid metabolites to lead to behavioral changes. Increases in urinary excretion of hippuric acid, a product of gut microbiota metabolism, were measured following 8 weeks of a high‐polyphenol diet,^[^ ^135^ ^]^ while increased excretion was detected after only 2 weeks following blueberry treatment in children.^[^ ^136^ ^]^ In addition to urinary metabolites, improvements to accuracy in a modified attention network task (MANT; a measure of executive function, attention, and inhibition) described in detail by Whyte et al.^[^ ^112^ ^]^ were also concurrently observed in the same study following 2 and 4 weeks of blueberry treatment.^[^ ^136^ ^]^ Clarity around the length of supplementation required for metabolic effects is therefore required, particularly across different age groups. Whether cognitive changes consistently coincide with changes in metabolites remains to be established.

In a recent commentary on published reviews of polyphenol effects on cognition, Lamport and Williams^[^ ^22^ ^]^ observed the reporting of more pronounced cognitive effects following chronic polyphenol interventions. This was attributed to the greater number of chronic studies reviewed, which is also reflected in the findings here. Additionally, acute effects can be masked by numerous, transient confounds such as recent diet, time of day of testing, and recent exercise. A possible interpretation is that acute effects are likely more subtle in nature, rendering them less detectable than chronic effects. The cognitive domains or tasks that are sensitive to acute treatment may also differ from those of chronic intervention. For example, faster reaction times were recorded in the MANT following acute blueberry supplementation^[^ ^54^ ^]^ compared to improved accuracy scores in the MANT following chronic blueberry supplementation.^[^ ^136^ ^]^

Our subgroup analysis for age of participants suggests flavonoids are particularly beneficial to cognition in middle‐aged and older adults. This may reflect the commencement of age‐related cognitive decline from middle age onward, thus increasing the likelihood of flavonoids exerting a detectable effect. Young adults at the peak of their cognitive ability may exhibit ceiling effects during testing leading to nonsignificant effects of flavonoid supplementation.^[^ ^137^ ^]^ Cognitive testing in future research should therefore account for this through careful selection of cognitive tasks with proven sensitivity in each age group. Significant positive outcomes were seen in a previous meta‐analysis of studies investigating young and middle‐aged adults.^[^ ^26^ ^]^ Our subgroup analysis suggests that this may have been driven by the effects seen in middle‐aged adults. As with our observations for middle‐aged adults, Cheng et al.^[^ ^29^ ^]^ showed soya isoflavones were effective in postmenopausal women younger than 60 years. However, the effects in women older than 60 years were nonsignificant. This may represent an effect that is specific to isoflavones. Indeed, our findings demonstrate a clear benefit of flavonoids more generally in older adults, which is consistent with previous findings.^[^ ^21^, ^25^ ^]^ Regarding children, only five studies were included in our meta‐analysis. All five studies investigated acute blueberry interventions meaning that the findings are not representative of flavonoid sources more generally, and do not inform us on potential chronic flavonoid effects in children. The nonsignificant outcome for this subgroup may be partially due to the limited number of studies (*k* = 5) coupled with the acute designs. The effects of flavonoids on cognition in children therefore remain unclear and warrants further research, particularly in foods outside of the berries group.

The subgroup analysis of cognitive health factors showed significant positive effects of flavonoids on cognition in both cognitively healthy and unhealthy subjects, but effect sizes were observed to be significantly greater for the latter population. This is likely due to greater scope for cognitive improvement in the cognitively unhealthy. These results are in keeping with meta‐analyses which have shown cognitive benefits of supplementing with gingko biloba in Alzheimer's disease and cognitive decline.^[^ ^125^, ^126^, ^127^, ^128^, ^138^ ^]^ Importantly, our findings show flavonoids are beneficial to the cognitive performance of cognitively unhealthy subjects, including patients with MCI and AD. This result is particularly pertinent in light of the increasing financial and social burden of neurodegenerative diseases in an aging population. The potential clinical significance of this outcome warrants further investigation. Furthermore, the potential for flavonoid supplementation to support the maintenance of cognition in healthy populations and prevent onset of neurodegenerative disease may in fact have greater impact than attempts to treat such conditions.

Analysis of the individual flavonoid dose categories showed significant positive effects on cognition following low and medium but not high doses. Low and medium dose studies comprised the majority of the analyzed studies. Thirteen studies used a high‐dose flavonoid intervention, representing 561 subjects. The reason for a lack of significant effects at high doses is unclear at this stage and warrants further investigation. The exact shape of the flavonoid‐cognition dose–response curve is yet to be established. Studies which have investigated a dose–response, comparing a range of high, medium, or low doses with a control, have demonstrated some benefits for higher doses (e.g.,^[^ ^17^, ^55^, ^61^, ^80^, ^85^, ^93^, ^99^, ^100^, ^105^ ^]^), indicating that the cognitive effects of flavonoids may increase with increasing dose, however these dose‐related effects are mainly restricted to cocoa and are yet to be fully investigated for other flavonoid sources. Indeed, possible reasons for this may be that high intakes are not recommended for some flavonoid sources such as ginkgo biloba, green tea, or soya due to the potential impact on the liver or endocrine system. Berries and citrus fruits may simply be difficult to consume in high amounts due to large volumes or side effects of high acidity and fibre. Methodologically, it should also be noted that while well‐designed dose response trials impart valuable information in isolation, the statistical constraints of a meta‐analysis preclude them from comparative subgroup analyses when combined with other single‐dose investigations. Therefore, as performed here, combined dose findings from such studies can only be investigated at the individual dose level. Irrespective of these issues, our findings have clear implications for potential public health messages in that the positive effects of flavonoids are observed from doses achievable within habitual daily dietary intake ranges.

Analysis of the individual cognitive domains showed significant beneficial effects of flavonoids on long term memory, mood, and processing speed. Cheng et al.^[^ ^29^ ^]^ adopted a similar methodology to examine the effects of soya isoflavones on different cognitive domains and found significant effects on visual memory only. In a comparative subgroup analysis Cui et al.^[^ ^30^ ^]^ showed significant effects of soya isoflavones on the memory domain,^[^ ^30^ ^]^ but also found that effect sizes were not significantly heterogeneous between domains, and therefore evidence of a particular domain showing greater sensitivity to flavonoid intervention was not demonstrated. Here, most studies included in this meta‐analysis adopted a battery of cognitive tasks which assessed a range of cognitive domains. As with dose, a comparative subgroup analysis to directly compare the effect of flavonoids on the different cognitive domains was therefore not possible. Further investigations into the relative sensitivity of cognitive domains to flavonoid intervention are needed. A consensus in the cognitive tasks adopted across intervention studies would help to achieve this by allowing the meta‐analysis of data for specific tasks.

With respect to the positive outcomes for long‐term memory and processing speed, these are likely to be products of the large number of studies included in the current meta‐analysis to assess performance in these domains. In relation to long‐term memory, this is unsurprising given the focus of the majority of cognitive‐flavonoid research has been placed on the older adult population, in which preservation or improvement of memory is of particular interest. Although memory appears to consistently respond to flavonoid supplementation, whether it shows greater sensitivity over other domains remains inconclusive. The lack of positive findings for the cognitive domains of word fluency, acquired knowledge, fluid reasoning, working memory/short‐term memory, visuospatial ability, and reaction/decision speed could be due to the relatively low number of studies in each subgroup, providing insufficient power to detect small effects. Additionally, it is also possible that the tasks employed to assess each of these domains may not have been sufficiently sensitive to detect subtle cognitive changes.

Finally, for the comparative analysis of crossover and parallel study designs, there was no significant heterogeneity observed between subgroups. Importantly, this addresses potential compatibility issues of including both types of design in a meta‐analysis and provides justification for the adopted methodology. This result suggests that any potential underweighting of effect sizes in crossover studies was minimal, with little impact on the overall estimated effect size.

### 4.2 Summary and Conclusions

This meta‐analysis provides evidence for the positive effects of flavonoids on cognitive function and demonstrates the moderating influence of a number of factors, including duration of supplementation and dietary source of flavonoid. It appears that for chronic effects, at least 6 weeks of supplementation may be required for benefits to manifest, and improvements may be more likely following either blueberry, cocoa, or ginkgo supplementation. The potential of these and other flavonoid‐rich foods to mitigate pathological cognitive decline in healthy individuals requires clarification in future research. Of the four comparative analyses performed, the only factor to demonstrate significant heterogeneity between subgroups was cognitive health status. Indeed, cognitive health status emerged as an important determinant of the magnitude of cognitive effects. While significant benefits were seen across both cognitively healthy and unhealthy populations, they were most apparent in the cognitively unhealthy, with pre‐existing cognitive deficits likely to facilitate the subsequent improvements seen in middle‐aged and older adults. Absence of heterogeneity of effect sizes between flavonoid sources, duration of supplementation, and age of participants subgroups could indicate a genuine absence of significant difference, or more likely, certain subgroups are under‐represented and, therefore, further high‐quality RCTs are required.

A strength of this meta‐analysis is the extensive coverage of the existing literature, incorporating a wide range of flavonoid sources, and populations, and allowing a detailed examination of a host of contributing variables. Reassuringly, there were few potential issues with bias in the literature, and the fail‐safe N for the global analysis was high allowing confidence in the findings. We are aware of some limitations to this review. Studies were restricted to those published in English. The cognitive effects observed have been interpreted in relation to the flavonoid content of the interventions described. However, it is acknowledged that nonflavonoid polyphenols may also have contributed to these outcomes.

There were also a low number of studies which investigated citrus, pine bark, and tea, or that investigated the effects of flavonoids on cognition in children outside of blueberry supplementation. Therefore, there is scope for further examination in these areas with appropriate controls. Indeed, this meta‐analysis highlights several areas where the number of quality studies is limited, culminating in several recommendations. Well‐designed dose–response studies are needed to establish optimal and dietary‐relevant flavonoid doses to better inform public health messages relating to flavonoid intake. It also remains unclear whether flavonoids exert their effects on specific cognitive domains, and so future research should aim for a consensus on the cognitive tasks used, facilitating consistent investigation across a broad spectrum of domains, and allowing easy comparison between studies in meta‐analysis or systematic review. Establishing domain‐specific effects would support better targeting of potential patient or consumer recommendations, for example, where cognitive health priorities may vary between age groups. Overall, the positive impact of flavonoid supplementation on cognitive health observed here presents the potential for a highly accessible, safe, and cost‐effective intervention program to tackle the burden of cognitive decline.

## Conflict of Interest

The authors declare no conflict of interest.

## Author Contributions

N.C. and L.B. contributed equally to this work. N.C. and L.B. designed the meta‐analyses, performed the study search, performed the meta‐analyses, and wrote the manuscript (equal contribution); D.L. and C.W. designed the meta‐analysis and critically revised the paper.

## Supporting information

Supporting Information

## Data Availability

The data that support the findings of this study are available on request from the corresponding author. The data are not publicly available due to privacy or ethical restrictions.
