# Supplementary material for: Dietary Flavonoids and Human Cognition: A Meta‐Analysis
Source: Mol Nutr Food Res. 2022 Apr 7;66(21):2100976. doi: 10.1002/mnfr.202100976 (PMC9787524; doi:10.1002/mnfr.202100976)
Supplement: Supplementary file 1 — Supporting Information [file MNFR-66-2100976-s001.pdf]

Supplementary Information

Table S1: Effect of flavonoids on cognition by flavonoid source

Flavonoid Source	k	Mean weighted g	95% CI (lower limit, upper limit)	p
**Berry**	23	0.149	0.038, 0.261	**0.009**
**Citrus**	3	0.065	-0.242, 0.371	0.679
**Cocoa**	11	0.224	0.014, 0.434	**0.036**
**Ginkgo**	22	0.187	0.103, 0.271	**<0.001**
**Pine Bark**	2	0.011	-0.347, 0.370	0.950
**Soya**	15	0.054	-0.055, 0.162	0.331
**Tea**	2	0.093	-0.228, 0.414	0.571
**Other**	2	0.119	-0.289, 0.527	0.568

Table S2: Effect of flavonoids on cognition by acute or chronic design

Acute or Chronic Study Design	k	Mean weighted g	95% CI (lower limit, upper limit)	p
**Acute**	31	0.094	-0.002, 0.190	0.055
**Chronic**	51	0.151	0.081, 0.221	**<0.001**

Table S3: Effect of flavonoids on cognition by duration of supplementation

Duration of flavonoid supplementation	k	Mean weighted g	95% CI (lower limit, upper limit)	p
**≤ 24 hours**	31	0.094	-0.002, 0.190	0.055
**> 24h ≤ 6 weeks**	3	-0.020	-0.367, 0.328	0.912
**> 6 weeks ≤ 3 months**	31	0.150	0.058, 0.243	**0.001**
**> 3 months**	19	0.154	0.040, 0.267	**0.008**

Table S4: Effect of flavonoids on cognition by participant age group

Age group	k	Mean weighted g	95% CI (lower limit, upper limit)	p
**Children**	5	0.171	-0.074, 0.416	0.172
**Young Adults**	24	0.088	-0.018, 0.194	0.105
**Middle-aged Adults**	22	0.112	0.007, 0.217	**0.037**

**Older Adults**	29	0.176	0.074, 0.278	**0.001**

Table S5: Effect of flavonoids on cognition by participant cognitive health status

Cognitive Health Status	k	Mean weighted g	95% CI (lower limit, upper limit)	p
**Healthy**	72	0.103	0.048, 0.158	**<0.001**
**Cognitively Unhealthy**	8	0.306	0.143, 0.469	**<0.001**

Table S6: Effect of flavonoids on cognition by flavonoid dose

Flavonoid Dose	k	Mean weighted g	95% CI (lower limit, upper limit)	p
**Low**	46	0.094	0.020, 0.168	**0.013**
**Medium**	31	0.162	0.092, 0.232	**<0.001**
**High**	13	0.100	-0.046, 0.245	0.181

Table S7: Summary of comparative analyses showing between subgroup heterogeneity

Moderator	Q	p
**Flavonoid Source**	Q(7) = 5.109	0.647
**Duration of flavonoid supplementation**	Q(3) = 1.604,	0.659
**Age group**	Q(3) = 1.605	0.658
**Cognitive Health Status**	Q(1) =5.319	**0.021**
